# Flow cytometry data standards

**DOI:** 10.1186/1756-0500-4-50

**Published:** 2011-03-07

**Authors:** Josef Spidlen, Parisa Shooshtari, Tobias R Kollmann, Ryan R Brinkman

**Affiliations:** 1 Terry Fox Laboratory, BC Cancer Agency, Vancouver, BC, Canada; 2 Department of Computer Science, University of British Columbia, Vancouver, BC, Canada; 3 Division of Infectious & Immunological Diseases, Department of Pediatrics, University of British Columbia, Vancouver, BC Canada; 4 Department of Medical Genetics, University of British Columbia, Vancouver, BC, Canada

## Abstract

**Background:**

Flow cytometry is a widely used analytical technique for examining microscopic particles, such as cells. The Flow Cytometry Standard (FCS) was developed in 1984 for storing flow data and it is supported by all instrument and third party software vendors. However, FCS does not capture the full scope of flow cytometry (FCM)-related data and metadata, and data standards have recently been developed to address this shortcoming.

**Findings:**

The Data Standards Task Force (DSTF) of the International Society for the Advancement of Cytometry (ISAC) has developed several data standards to complement the raw data encoded in FCS files. Efforts started with the Minimum Information about a Flow Cytometry Experiment, a minimal data reporting standard of details necessary to include when publishing FCM experiments to facilitate third party understanding. MIFlowCyt is now being recommended to authors by publishers as part of manuscript submission, and manuscripts are being checked by reviewers and editors for compliance. Gating-ML was then introduced to capture gating descriptions - an essential part of FCM data analysis describing the selection of cell populations of interest. The Classification Results File Format was developed to accommodate results of the gating process, mostly within the context of automated clustering. Additionally, the Archival Cytometry Standard bundles data with all the other components describing experiments. Here, we introduce these recent standards and provide the very first example of how they can be used to report FCM data including analysis and results in a standardized, computationally exchangeable form.

**Conclusions:**

Reporting standards and open file formats are essential for scientific collaboration and independent validation. The recently developed FCM data standards are now being incorporated into third party software tools and data repositories, which will ultimately facilitate understanding and data reuse.

## Findings

In FCM, intact cells and their constituent components are tagged with fluorescently conjugated antibodies and/or stained with fluorescent reagents and then analyzed individually. In a flow cytometer, cells in suspension are excited by a laser and the fluorescence emission from each cell is collected by a series of photomultiplier tubes. Subsequent electrical events are collected and analyzed on a computer that assigns a fluorescence intensity value to each signal. These values are stored in the Flow Cytometry Standard (FCS) data file format developed by ISAC in 1984 and it is still the common representation of FCM data supported by all instruments and FCM data analysis tools [1]. FCS was recently extended to version 3.1 [2], correcting some ambiguities, improving support for international characters and storing compensation, and adding support for preferred display scale, sample volume, tracking originality of data files and plate and well identification.

While FCS is the essential data standard in FCM, and is supported by all instrument vendors and third party software tools, it does not capture the protocol used or the computational post-processing and data analysis performed in an FCM experiment. Recently, ISAC's Data Standards Task Force (DSTF) developed additional file formats capturing details about FCM data analysis as required by the Minimum Information about a Flow Cytometry Experiment (MIFlowCyt, [3]).

## Gating-ML File Format

In FCM, the process for selecting populations of interest is known as gating. This process is traditionally conducted manually by drawing boundaries around cell populations, and this activity is supported by all FCM analysis software applications. However the definition of the boundaries has not been exchangeable between software tools, which are each storing this information in their own specific, often binary format. The lack of software interoperability on the gating level is a major bottleneck preventing independent reproducibility of FCM data analysis, usage of multiple analytical tools, and development of novel analytical and clinical methods. A standard formal way of exchanging unambiguous descriptions of gates is crucial for interoperability among analytical hardware and software applications. To address this need, the Extensible Markup Language (XML) [4] was chosen as a simple, open, flexible, self-describing, properly established, widely supported, and therefore well suited technology. In 2008, ISAC's DSTF developed an initial specification of Gating-ML [5] - an XML-based file format accommodating exact and reproducible descriptions of gates. The Gating-ML specification is increasingly supported by both commercial software vendors (e.g., TreeStar Inc., makers of FlowJo, Cytobank Inc.) as well as open source software, such as the flowCore/flowUtils [6] R/BioConductor [7] packages. With these reference implementations now available, the Gating-ML Candidate Recommendation is under revision as a first version of the official ISAC standard for computational description of gates.

## Classification Results File Format

Recently, the increased amount of high-throughput and high-content FCM data motivated the development of various automated methods to supplement manual gating. Similar to manual gating, the results of these methods are commonly per-event-based, potentially fuzzy, classifications expressed as the probability of a cell being a member of a class of cells. Unlike manual gating, the geometrical boundaries of these automatically derived classes are often difficult or even impossible to define, which prevents or complicates the use of Gating-ML. The Classification Results (CLR [8]) File Format is currently being finalized to address this issue. While Gating-ML contains mathematical definitions of gates, the CLR format only captures results of the gating process (i.e., the assignment of cells to classes of cells (e.g., cell types)). Consequently, the CLR format is usable for the results of both manual and automated gating. While XML or similarly expressive technologies could have been utilized for this purpose, a simpler format is sufficient and essentially beneficial for end users in this case. Therefore, the CLR specification has been based on the common CSV [9] file format and consequently, a CLR file can be easily parsed, and can be created and viewed using commonly available spreadsheet software.

## Archival Cytometry Standard File Format

The Archival Cytometry Standard (ACS) was developed to bundle data with all the other components describing experiments. Besides capturing relations among data and other files, the requirements for such a file format included the support for audit trails, versioning and digital signatures. Several alternatives have been considered. For example, RDF [10] and related semantic web technologies have been evaluated in terms of capabilities to capture experimental work flows. While very promising, these technologies are still evolving and they do not have a stable support in existing tools. Therefore, the Data Standards Task Force chose a more conservative approach based on widely-accepted tools and technologies. As a result, the ACS container is based on the ZIP file format with an XML-based Table of Contents specifying relations among files in the container. Since ACS is based on the ZIP file format and ZIP is supported on virtually all platforms, it is possible to create ACS containers without specialized software tools. In addition, W3C XML Signatures [11] have been incorporated to facilitate the audit trail with appropriate digital signatures. Similar to the other mentioned formats, ACS is increasingly being supported by commercial and open source software and data repositories.

## Example Dataset

**Figure 1:**
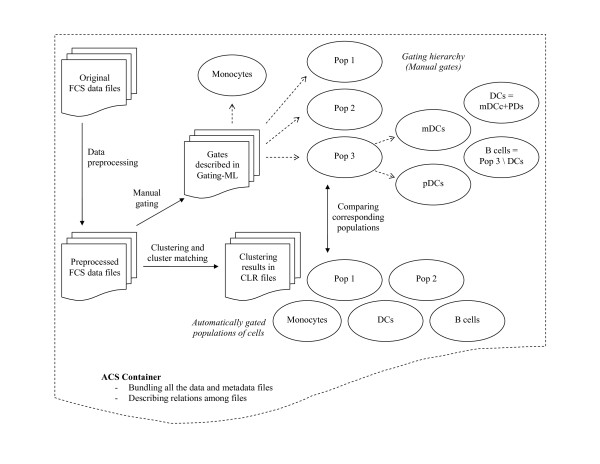
**Analytical pipeline captured in the provided ACS example file**. The Figure shows an overview of the analytical pipeline captured in the ACS example file. The work flow is demonstrated by solid line arrows. Data files are preprocessed and manually gated for monocytes, dendritic cells (DCs, including myeloid and plasmacytoid subpopulations - mDCs and pDCs) and B cells, as well as additional novel populations whose roles in the immune response still need to be further investigated. The gating hierarchy (stored in Gating-ML files) is indicated by dashed line arrows. In parallel to this effort, the preprocessed FCS data files are fed to a clustering and cluster matching algorithm, which identifies monocytes, dendritic cells, B cells and additional subpopulations independently of the manual gating. The results of the automated and manual gating process are compared in order to determine the suitability of this automated analysis approach.

We provide the first example of how these recently developed standardized file formats can be used to report FCM data including analysis and results in a standardized, computationally exchangeable form within an ACS container (see Additional file 1). The ACS container captures data and an analytical pipeline as shown in Figure 1. The ACS container includes two FCS data files acquired on an LSR II cytometer as part of an immune response study, two preprocessed FCS data files, two Gating-ML files capturing the process of manual gating, two CLR files with results of automated clustering, and an XML-based table of contents capturing relations among all the components. The original FCS files are randomly chosen from a set of FCM data samples of peripheral blood mononuclear cells (PBMC). Each sample is stimulated by a specific microbe-derived danger signal at a specific concentration in order to investigate in cellular detail the recognition and responses of human antigen presenting cells (APCs) to the danger signals of interest. APCs form a major group of cells in the immune system responsible for detecting foreign microbes and presenting them to other immunity cells for further actions. Subpopulations of APCs include monocytes, plasmacytoid dendritic cells (pDC), and myeloid dendritic cells (mDC), each of which differ in functionality as they respond differently when challenged with the same danger signals. APCs respond to danger signals in a number of ways, one of which is through secretion of cytokines. Cytokines are cell-signalling molecules that are used in intercellular communications, and can activate other immune cells to take further actions against microbes and pathogens.

Our example FCS data files contain measurements corresponding to physical characteristics of the cells (i.e., forward and side scatter), cell surface proteins (CD123, CD11c, MHC-II, CD14), and intracellular cytokines (IL-6, IL-12, TNF-*α*, IFN-*α*). During the analysis, an initial series of preprocessing steps were applied to the data. This included the removal of dead cells and debris, compensation and subsetting to the desired channels. Then the cell surface proteins measurements of the preprocessed FCS files are used to gate the files manually with the purpose of identifying cell populations including monocytes, B cells, and dendritic cells (DCs, including myeloid and plasmacytoid subpopulations - mDCs and pDCs), as well as two additional novel populations, whose roles in the immune response still need to be further investigated. Parallel to the manual approach, the preprocessed FCS files were analyzed using the SamSPECTRAL [12] clustering algorithm, followed by a semi-automated cluster matching technique to identify the desired populations automatically. Populations identified by automated clustering were compared to manually gated populations in order to determine the suitability of this automated approach in identification of cell populations of interest. Information corresponding to the results of these analyses were captured in a computer processable form in the ACS container.

A combination of tools was used to create the ACS container and it's components. Compensation and the live cell gates were created in FlowJo. The spillover matrix and gate coordinates were exported from FlowJo and used in the flowCore [6] R/BioConductor [7] package in order to preprocess the FCS data files. The preprocessed FCS files were manually gated in FlowJo and the gates were exported in Gating-ML. In parallel, the SamSPECTRAL [12] clustering algorithm was applied on the preprocessed data and resulting clusters were labelled and the results were saved in the CLR file format using the write.table R function. All files were manually organized in file system folders in an ACS-like structure and a table of contents was created according to the ACS specification. The oXygen software tool (Syncro Soft Ltd.) was used to adjust and validate XML-based components of the container (i.e., the table of contents and the Gating-ML files). Finally, the ACS-like folder structure was zipped using Ubuntu's built-in compression support (any ZIP-compatible tool could have been used) and the file extension of the resulting file was changed to .acs as required by the ACS specification.

## Conclusions

Reporting standards and open file formats are essential components allowing for scientific collaboration and independent validation. The purpose of publishing this small subset of the data is to demonstrate the use of novel open file formats to computationally describe the analysis of FCM data. As it is often the case with new standards, their adoption requires investments at the beginning that will result in benefits down the road. In FCM, several leading software and hardware vendors have already incorporated support for the recently developed standards and therefore, these novel file formats are now effectively coming to the flngertips of researchers and other end users. Having the raw data along with the mechanism of analysis and results provided in a machine processable description makes the analysis reproducible, which is usually not the case with only figures and free text descriptions provided in most manuscripts [13]. Unfortunately, even with appropriate data and meta data standards in place, due to the lack of a public repository there is still a bottleneck preventing FCM data to be efficiently reusable. In order to address this issue, ISAC is currently developing a public data repository that will allow for annotated FCM data to be openly shared, which will ultimately facilitate third-party understanding and reuse of these data. This repository will support all relevant standards and will allow researchers to deposit annotated FCM data and associated analysis files along with MIFlowCyt-compliant textual descriptions of experiments.

## Availability and Requirements

**Project name: **Bioinformatics Standards for Flow Cytometry

**Project home page: **http://flowcyt.sourceforge.net

**Project mailing lists: **http://sourceforge.net/mail/?group_id=175725

**Operating systems: **Platform independent

**Programming languages: **Language independent; XML, ZIP and CSV processing libraries essential for developers to support mentioned standards.

**Other requirements: **None

**License: **Creative Commons Attribution-ShareAlike 3.0 Unported

**Any restrictions to use by non-academics: **None in addition to the above open source license agreement.

## Competing interests

The authors declare that they have no competing interests.

## Authors' contributions

JS led the development of ACS, CLR, Gating-ML and FCS 3.1 standards under the auspices of ISAC's DSTF. He wrote the initial version of the manuscript and created the presented example. PS analyzed the example data, provided biological insights and helped prepare the manuscript. TK provided the example data and helped prepare the manuscript. RRB is the Chair of ISAC's DSTF and helped prepare the manuscript. All authors reviewed and approved the final version of the manuscript.

## Supplementary Material

Additional file 1 **Archival Cytometry Standard example**. Archival Cytometry Standard (ACS) files are zip-compliant and can be opened with any tool that can process zip files. Click here for file
